# Maturation state of colonization sites promotes symbiotic resiliency in the *Euprymna scolopes-Vibrio fischeri* partnership

**DOI:** 10.1186/s40168-023-01509-x

**Published:** 2023-03-31

**Authors:** Tara Essock-Burns, Susannah Lawhorn, Leo Wu, Sawyer McClosky, Silvia Moriano-Gutierrez, Edward G. Ruby, Margaret J. McFall-Ngai

**Affiliations:** 1 Kewalo Marine Laboratory, Pacific Biosciences Research Center, University of Hawai’i, Mānoa, Honolulu, HI USA; 2 Present address: Carnegie Institution for Science, Division of Biosphere Sciences and Engineering, Pasadena, CA USA; 3 Present address: Department of Fundamental Biology, University of Lausanne, Lausanne, Switzerland

**Keywords:** *Euprymna scolopes*, *Vibrio fischeri*, Microbiota, Antibiotic, Chloramphenicol, Symbiosis, Dysbiosis, Microbiome

## Abstract

**Background:**

Many animals and plants acquire their coevolved symbiotic partners shortly post-embryonic development. Thus, during embryogenesis, cellular features must be developed that will promote both symbiont colonization of the appropriate tissues, as well as persistence at those sites. While variation in the degree of maturation occurs in newborn tissues, little is unknown about how this variation influences the establishment and persistence of host-microbe associations.

**Results:**

The binary symbiosis model, the squid-vibrio (*Euprymna scolopes*-*Vibrio fischeri*) system, offers a way to study how an environmental gram-negative bacterium establishes a beneficial, persistent, extracellular colonization of an animal host. Here, we show that bacterial symbionts occupy six different colonization sites in the light-emitting organ of the host that have both distinct morphologies and responses to antibiotic treatment. *Vibrio fischeri* was most resilient to antibiotic disturbance when contained within the smallest and least mature colonization sites. We show that this variability in crypt development at the time of hatching allows the immature sites to act as a symbiont reservoir that has the potential to reseed the more mature sites in the host organ when they have been cleared by antibiotic treatment. This strategy may produce an ecologically significant resiliency to the association.

**Conclusions:**

The data presented here provide evidence that the evolution of the squid-vibrio association has been selected for a nascent organ with a range of host tissue maturity at the onset of symbiosis. The resulting variation in physical and chemical environments results in a spectrum of host-symbiont interactions, notably, variation in susceptibility to environmental disturbance. This “insurance policy” provides resiliency to the symbiosis during the critical period of its early development. While differences in tissue maturity at birth have been documented in other animals, such as along the infant gut tract of mammals, the impact of this variation on host-microbiome interactions has not been studied. Because a wide variety of symbiosis characters are highly conserved over animal evolution, studies of the squid-vibrio association have the promise of providing insights into basic strategies that ensure successful bacterial passage between hosts in horizontally transmitted symbioses.

Video Abstract

**Supplementary Information:**

The online version contains supplementary material available at 10.1186/s40168-023-01509-x.

## Introduction

In horizontally transmitted symbioses, the organ systems of the host, e.g., the mammalian gastrointestinal and respiratory tract, and the roots of leguminous plants, recruit their microbial partners from environmental reservoir populations [1–3]. Such symbiotic associations usually establish as stable partnerships immediately following embryogenesis [4–7] and, in animals, the microbes often take up residence as extracellular colonizers of the apical surfaces of polarized epithelia. Recent studies of a variety of symbiotic associations have shown that these dynamic relationships have effects on the form and function of both local and adjacent tissues in the associated organ system [8–12]. A variety of environmental disturbances, such as treatment with antibiotics or other drugs, changes in osmotic pressure, or predation by phage, can perturb a host’s interaction with its microbial populations and, thereby, detrimentally affect host health [13–16]. Such environmental disturbances can be particularly potent during early development of the host, and can even trigger a dysregulated microbiome, or dysbiosis. A dysbiotic microbiome can limit microbial metabolites, allow pathogen invasion and outgrowth, and trigger inflammation in the host [17, 18]. One widespread disturbance is the use of antibiotics, which, when introduced in early life can, later in life, lead to adverse health outcomes, such as obesity [19, 20], colitis [21, 22], increased susceptibility to autoimmune diseases such as asthma [23], and neuroinflammation and neurodegenerative disease [24–27]. A feature that influences the stability of symbiotic systems is the architecture and spatial heterogeneity of the associated tissues [28]. Physicochemical differences across these tissue microenvironments shape the microbial community at each site [28–31]. For example, within the mammalian gastrointestinal (GI) tract, the composition and metabolic activity of microbial assemblages, and their proximity to host tissue, differ across different microenvironments [32–36]. As a result, when the gut microbiome is destabilized following an environmental disturbance (e.g., [14]), recovery can be mediated by the recruitment of nearby microbial populations (e.g., [37, 38]).

**Fig. 1:**
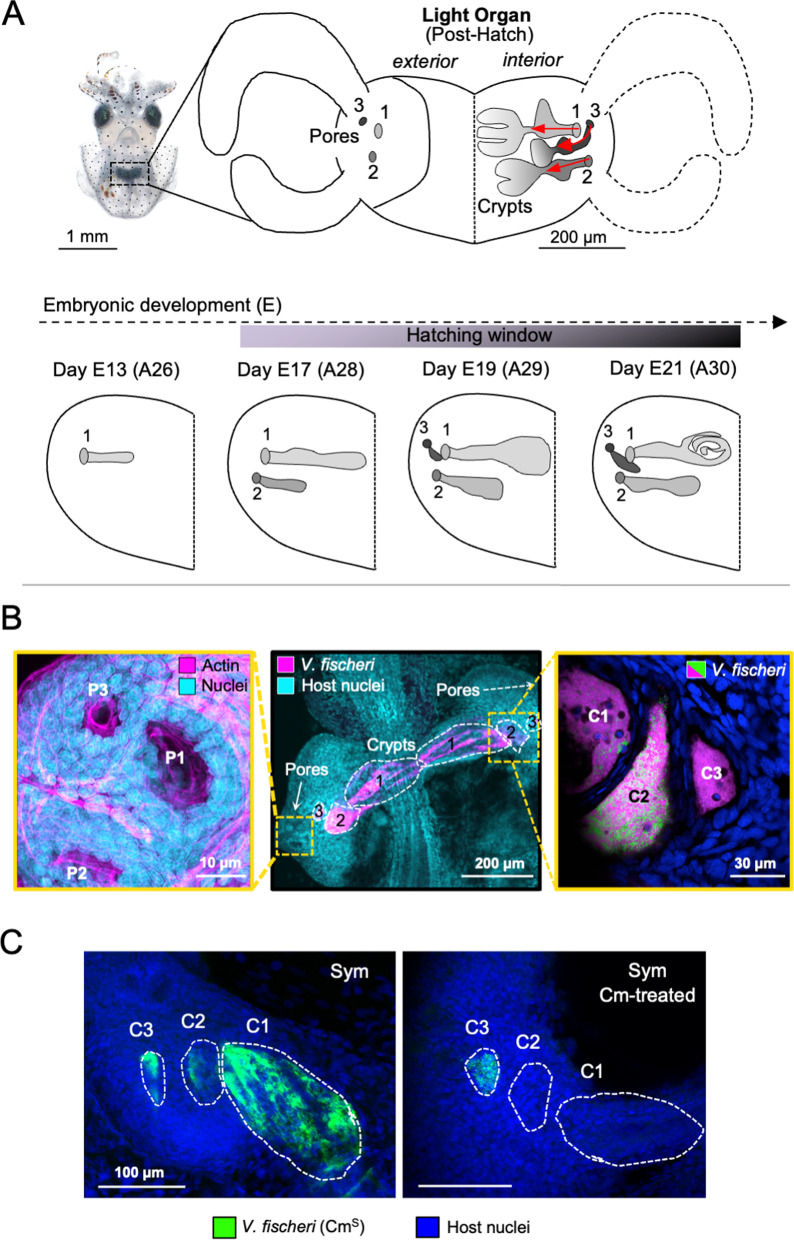
Overview of symbiotic host tissue—development of the squid light organ and key sites for interaction with bacteria. **A** *Left*, a hatchling *Euprymna scolopes* and its ventral light organ (black, dashed box). *Right*, schematic of the bilobed organ with three external pores per lobe. *V. fischeri* cells enter these pores and migrate down paths (red arrows) that lead to three independent crypts. *Bottom*, schematic of the formation of the interior symbiotic tissues over embryonic development on one half of the light organ; embryological day and standardized embryonic stage are based on [41]. **B** Confocal micrographs: *Left*, a high magnification of the three bacterial entry points or pores (P1-P3); *Center*, the bilobed light organ after full colonization by *V. fischeri* (magenta); *Right*, three crypts (C1-C3) of a single lobe showing mixed colonization (green and magenta) in the C1 and C2 crypts. **C** Confocal micrographs showing the crypts of one lobe of the light organ colonized for 24 h by the Cm-sensitive (Cm.^S^) and GFP-labeled, wild-type strain (i.e., strain ES114 carrying pVSV102; see Table 1); then, for 24 h, either untreated (left) or treated with ≥50 μM Cm (right)

While resiliency of animal-microbe partnerships following antibiotic pressure has been extensively studied under a variety of circumstances, the mechanisms by which events in embryogenesis participate in promoting the stability of initial host-symbiont interactions are poorly understood. The early dynamics of symbiotic development can be difficult to characterize: the tissues are often inaccessible and critical events occur across time frames that limit the resolution of the analyses. The model symbiosis between the Hawaiian bobtail squid, *Euprymna scolopes*, and its luminous bacterial partner, *Vibrio fischeri*, offers an experimentally tractable association for the study of early events in microbiome development (Fig. 1; for review see [39]). During the second half of embryogenesis, the nascent light-emitting organ develops as a lateral proliferation of cells in the region of the hindgut [40]. In this process, three crypt spaces (designated C1, C2, C3), which will be the eventual sites of symbiont colonization, arise in sequence on each side of the developing organ as invaginations of the surface (Fig. 1A). The first invagination, which produces C1, begins at two thirds of the way through the embryonic period, which averages 21 days [40]. The second (C2) begins ~ 4 days later and the third (C3) begins just 2 days before hatching. This staggered process of appearance creates crypts of varying maturity. Further, variation in crypt maturity also depends upon both the genetic background of the hatchlings as well as the timing of hatching [42]. Such variation most strongly impacts the size and shape of C3 at hatching. Embryogenesis also results in a specific arrangement of the crypts relative to one another, which is independent of the maturation state of any of the crypts [40, 42].

Recruitment of symbionts to the light organ begins within minutes of the host squid’s hatching into the seawater environment. Symbionts aggregate on the surface of the organ and, depending on the strain of *V. fischeri*, enter host tissues in as little as 15 min, ultimately coexisting at least transiently as mixed-strain populations within individual crypts [43]. From the superficial pores, they travel along a ~ 150-μm migration path to reach the crypts (Fig. 1A, top right), where they proliferate to fill the crypt spaces and begin to luminesce by ~ 12 h (for reviews, see [39, 44]). The metabolically active symbionts interact both among themselves within a given crypt, and with the host cells that support their populations [45–47]. Through the diffusion of soluble materials, the symbionts also communicate with *V. fischeri* cells contained in the other crypts [48].

The three crypts on each side of the light organ, however, differ in several ways: (i) the colonizing symbionts in each of the two C3 are slower in the onset of luminescence [49] than those in the other crypts; (ii) they are a greater distance from the apical surface of the host epithelial cells that line the crypts [50]; (iii) they do not cause the typical symbiont-induced swelling of the crypt epithelial cells [42]; (iv) symbiont cells with compromised viability are detected in C3, but not in C1 or 2; (v) in the early days of the symbiosis, C3 does not undergo the typical expulsion of most of its symbionts into the surrounding seawater, a behavior that occurs each day at dawn, beginning with the first day of symbiosis [49–51]; and (vi) standardized protocols for antibiotic clearing (chloramphenicol) of the organ result in the loss of symbionts in C1 and 2, but not 3 [51].

Because of the observation that the symbionts in C3 of the newly colonized juvenile light organ are resistant to antibiotic treatment, in this study, we sought to explore the role of tissue microbiogeography in the resilience of a symbiosis to disturbance during the early hours to days following the onset of the partnership. We find that the tissue landscape produced during embryogenesis creates within C3 a reservoir of symbionts that provides resiliency to the newly established partnership. Because the persistent association of microbes along the surfaces of epithelia is highly conserved throughout the animal kingdom, it is likely that similar strategies of varying microbiogeography provide resilience in other horizontally transmitted symbioses.

## Materials and methods

### Bacterial strains, plasmids, and growth conditions

The two isogenic strains used in this study were both *Vibrio fischeri* ES114 carrying one of two different plasmids. The wild-type *V. fischeri* strain carrying a green fluorescent protein (GFP) marker was sensitive to chloramphenicol (Cm) (*Vf* Cm^S^). A derived *V. fischeri* strain carrying resistance to Cm (*Vf* Cm^R^) was tagged with a red fluorescent protein (RFP) (see Table 1) [49]. These *V. fischeri* were cultured in Luria–Bertani salt (LBS) medium (10 g of Bacto-Tryptone, 5 g of yeast extract, 20 g of NaCl, and 50 mL of 1 M Tris–HCL buffer [pH 7.5] per liter of deionized water) as described previously [52]. LBS cultures were shaken at 225 rpm and 28 °C overnight and then diluted 1:1000 in Saltwater Tryptone (SWT) medium (5 g of Bacto-Tryptone, 3 g of yeast extract, 3 mL of glycerol, 700 ml of FSW, and 300 ml of distilled water) [47]. SWT subcultures were grown to mid-log phase and then diluted to an OD_600_ (optical density at 600 nm) of 0.2 before inoculating seawater containing hatchling squid. To maintain selection pressure on each strain carrying a plasmid, the appropriate concentration of antibiotic was added to the LBS overnight cultures: 2.5 μg mL^−1^ Cm for the Cm^R^, GFP-labeled strain; and 100 μg mL^−1^ kanamycin (Kn) for the strain carrying the Kn^R^ plasmid pVSV102, used as the Cm^S^, RFP-labeled strain. For the plating assays described below, LBS agar medium was prepared by the addition of 1.2% Bacto-Agar.

**Table 1:** Strains used in the study

Strain	Description	Phenotype	Reference
ES114	*E. scolopes* light organ symbiont *Vibrio fischeri*	Wild type	(51)
ES114 pVSV102	Strain tagged with *gfp* and Kn resistance	Cm^S^	(48)
ES114 pVSV208	Strain tagged with *rfp* and Cm resistance	Cm^R^	(48)

### In vitro characterization of antibiotics 

**Fig. 2:**
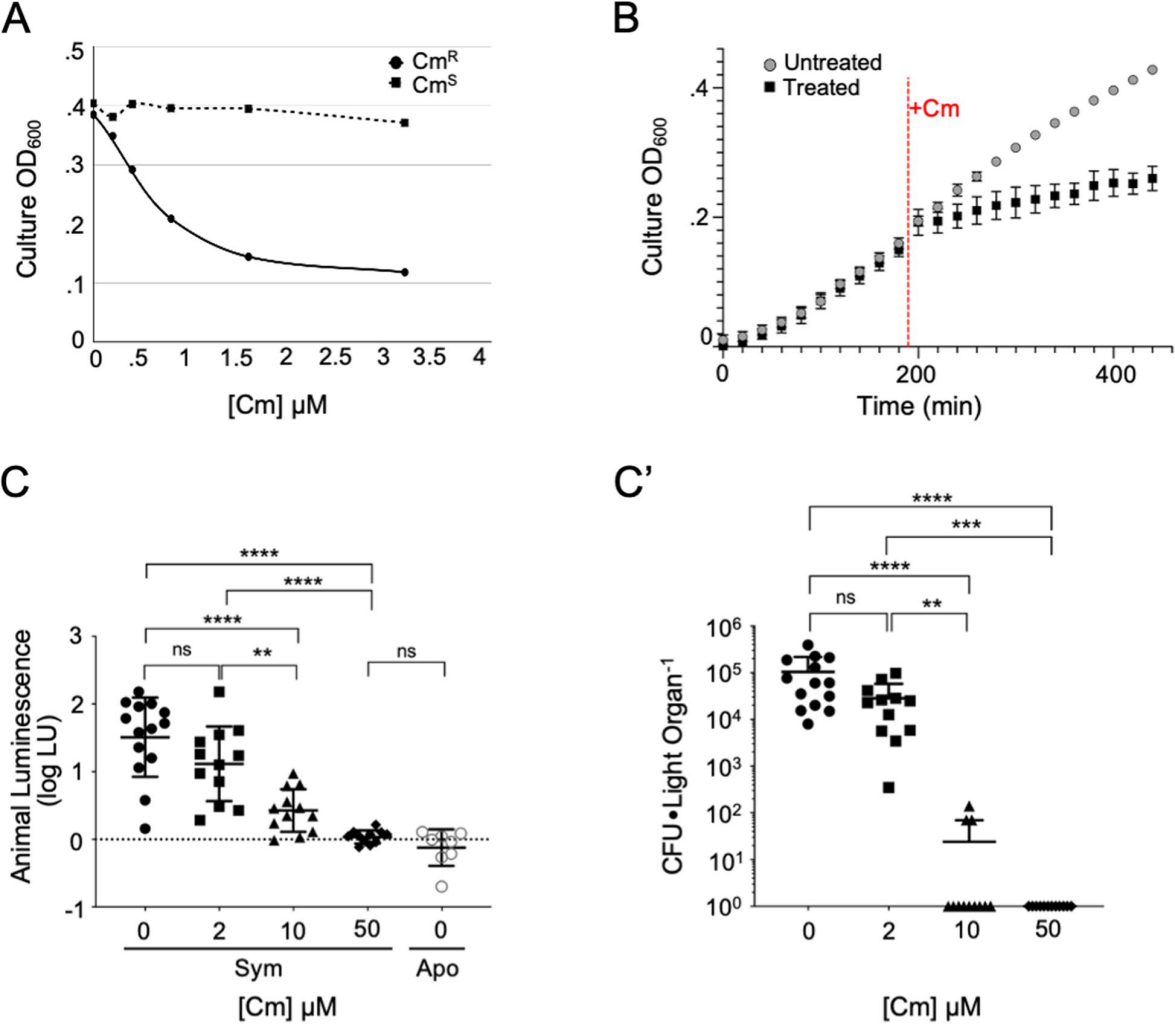
*V. fischeri* sensitivity to antibiotic treatment in culture and in the host. **A** Endpoint absorbance in SWT of either Cm^R^ or Cm^S^ *V. fischeri* strains, after a 2-h incubation in culture with various concentrations of Cm (data shown are representative of two trials). **B** Effect of the addition of 0.8-μM Cm on the growth of Cm^S^ *V. fischeri* in SWT; time added, red dashed line. Slope of the line (*Y* = 0.0002927*X + 0.1334) for the treated group was reduced to 30% of the untreated control and was statistically different from zero (*F*_1,11_ = 844.6, *P* $<$ 0.0001). **C** Luminescence of animals colonized by Cm^S^ *V. fischeri* for 48 h, with incubations in various concentrations of Cm for the second 24 h (*n* = 14, 7 animals from two replicate clutches; Apo, aposymbiotic control; dashed line, limit of detection). A Kruskal–Wallis test was used to analyze the effect of Cm on symbiont light output, H_(4,48)_ = 33.54, *P* $<$ 0.0001). **C’** Average symbiont number (CFUs) in each of these animals (threshold of detection = 1). A Kruskal–Wallis test was used to analyze the effect of Cm on symbiont number, H_(4,48)_ = 39.19, *P* $<$ 0.0001). For both C and C’, asterisks indicate significantly different values as determined by a Dunn’s multiple comparison test as follows: **** *P* $<$ 0.0001, *** *P* $<$ 0.001, ** *P* $<$ 0.01, ns = not significant

Growth kinetics of bacterial strains in vitro were measured using a SpectraMax iD5 microplate reader (Molecular Devices, San Jose, CA). For absorbance assays, cells grown from overnight LBS cultures were pelleted, washed, and diluted in SWT to an OD_600_ value of 0.05. SWT dilutions were added to 1 mL of fresh SWT in a 24-well microplate (Cat No. 08–772-51, Fisher Scientific, Hampton, NH). Assays were run in triplicate using cultures set up with distinct inoculum levels, defined as colony-forming units (CFUs) per mL. The program ran for 4 h at 28 °C with continuous shaking between readings. Luminescence and absorbance data were obtained every 20 min. Chloramphenicol (Cm) (Cat No. C0378, Sigma) was dissolved at RT on a rocker in filtered seawater (FSW; 0.22-μm pore-size). To assess the effect of Cm on *V. fischeri*, strains were grown in culture with various concentrations of Cm (Fig. S1), including doses comparable to what was used for treatment of animals. Because treatment with $\ge$ 3.3 μM (1 μg mL^−1^) Cm completely inhibited growth of the Cm^S^ strain (Fig. S1A), subsequent experiments in culture were done with $\le$ 3.3 μM Cm (Fig. 2A, B).

### Squid colonization

A breeding colony of adult *Euprymna scolopes*, collected from Paikō Bay of O’ahu, Hawaii, was maintained in running seawater tables at the Kewalo Marine Laboratory, Pacific Biosciences Research Center, University of Hawai’i at Mānoa, as previously described [53]. Clutches of eggs were collected and maintained in a flow-through egg table and kept on a 12:12 light/dark cycle. Hatchling squid were collected within 1 h of dark and cohorts from single clutch were used for each replicate experiment. To maintain squid aposymbiotically, squid were kept in Hawaiian offshore seawater (HOSW) without the addition of *V. fischeri*. To render the animals symbiotic, hatchlings were inoculated with *V. fischeri* cells at a concentration of 5 × 10^3^ CFU mL^−1^ in HOSW. To monitor colonization status at each time point, the animal’s luminescence was measured by a TD 20/20 luminometer (Turner Designs, Inc., Sunnyvale, CA) [54]. Luminescence readings $\le$ 1 were considered non-luminescent (dotted line on luminescence graphs) as this is the background luminescence detected for aposymbiotic animals.

### Antibiotic treatment and colonization level estimation

After 24 h of colonization, luminescent animals were incubated in FSW containing the various concentrations of Cm (Fig. 2C). The high dosage, referred to as $\ge$ 50 μM, used for squid experiments was either 50 μM (16 μg mL^−1^) or 62 μM (20 μg mL^−1^). The latter dosage has been used previously [55, 56], but a concentration as low as 31 μM (10 μg mL^−1^) for as short as 6 h has been previously shown to effectively clear the light organ of recoverable CFUs [53]. For experiments examining symbiotic recovery, after 24 h of Cm treatment, squid were rinsed by passage through FSW three times for 5 min each, before placing them in FSW.

To test whether Cm pressure affected the venting behavior of C3, we delivered a shorter pulse of a Cm disturbance before the animals’ second dawn. The Cm concentration for venting experiments was 20 μM (6.5 μg mL^−1^) and was delivered 6 h prior to the dawn light cue. Animal luminescence was measured before Cm exposure and then 30 min after the light cue, with and without Cm treatment.

Symbiont population levels in colonized animals were estimated by plating serial dilutions of homogenates of frozen animals, and counting the CFUs arising on LBS medium, as described previously [57]. The fluorescence of the CFUs (GFP or RFP) was determined to confirm the actual strain inoculation levels using a Stereo Microscope Fluorescence Adapter (NightSea, Electron Microscopy Sciences, Lexington, MA).

### Sample preparation and microscopy

To prepare samples for microscopy, juvenile squid (between 0 and 96 h post-hatch) were transferred to 4% paraformaldehyde in marine phosphate-buffered saline (mPBS; 50 mM sodium phosphate buffer, 450 mM NaCl, pH 7.4) and fixed overnight at 4 °C with rotation. Fixed samples were then washed three times for 30 min in mPBS prior to removal of the light organ by dissection. Light organs were permeabilized and stained in 0.1% Triton X-100 in mPBS for 2 days in the dark at 4 °C on an orbital rocker.

The nuclei were stained with TO-PRO-3 Iodide (ThermoFisher Scientific Cat No. T3605) diluted 1:1000 (excitation/emission [Ex/Em], 642/661 nm) and F-actin was stained with phalloidin (1:40 dilution) conjugated to either Alexa 405 (Ex/Em, 405/450 nm) or rhodamine (Ex/Em, 540/565 nm) (ThermoFisher Scientific Cat No. R415). For experiments described in Fig. S2, a fixable stain for dead cells was incubated with the animal for 6 h prior to fixation, using Live-or-Dye NucFix Red (Ex/Em, 520/593 nm) (Biotium, Fremont, CA) as described previously [50]. Once excess dye was washed off of fixed samples with mPBS, stained samples were mounted in Vectashield (Vector Laboratories, Burlingame, CA) and overlaid with a coverslip (number 1.5, Fisherbrand; Fisher Scientific, Waltham, MA) [50]. The laser scanning confocal microscopy was performed using an upright Zeiss LSM 710 microscope (Carl Zeiss AG, Jena, Germany), located at the University of Hawai’i at Mānoa, Kewalo Marine Laboratory. Images were acquired with a 1024 × 1024 format size and a 40 × oil objective, 1.4 NA, which yielded 0.3- to 0.8-μm resolution, depending on the optical zoom.

### Analyses

Confocal micrographs were analyzed using Fiji (ImageJ) for measurements and generations of projections of stacks [58]. A single lobe of a light organ was counted an individual set of crypts (C1-C3). Diameter of the bottleneck tissue was determined from confocal micrographs drawing a line at the narrowest point between the terminal web (F-actin staining) in a cross-section of the bottleneck as described previously [42, 50]. Distance between pores was measured as the smallest distance between the terminal web (F-actin staining) in a single plane at the superficial surface. The colonization status for each crypt was assigned a value as follows: 0 = 0 cells, 0.25 = 1–10 cells, 0.5 = 10–100 cells, 0.75 = 100–500 cells, 1 = 500 + cells. A crypt was considered “fully colonized” with a score of 0.75 or 1 and “uncolonized” with a score of 0. Venting efficiency was determined by the same operational definitions, but here, the number of symbiont cells in the migration path tissues connected to crypt 3 was scored by confocal microscopy. Statistical analyses were performed using GraphPad Prism software, version 9.0 (GraphPad Software, Inc., San Diego, CA).

Using Prism, the normality of all data was first tested with a D’Agostino and Pearson test and Kolmogorov–Smirnov test. Data that were considered normal were then analyzed by non-parametric tests such as a *t*-test, one-way ANOVA, or two-way ANOVA. Multiple comparisons were tested with a Tukey’s multiple comparison’s test. Non-normal data were log-transformed and retested. If data did not pass the normality test, then they were compared with parametric tests such as a Kruskal–Wallis and Dunn’s multiple comparison test. A chi-square was used to compare the frequency that each crypt type was colonized by the categories: primary strain, secondary strain, or mixed.

## Results

### Host-associated *Vibrio fischeri* cells withstand antibiotic treatment that inhibits growth in culture

**Fig. 3:**
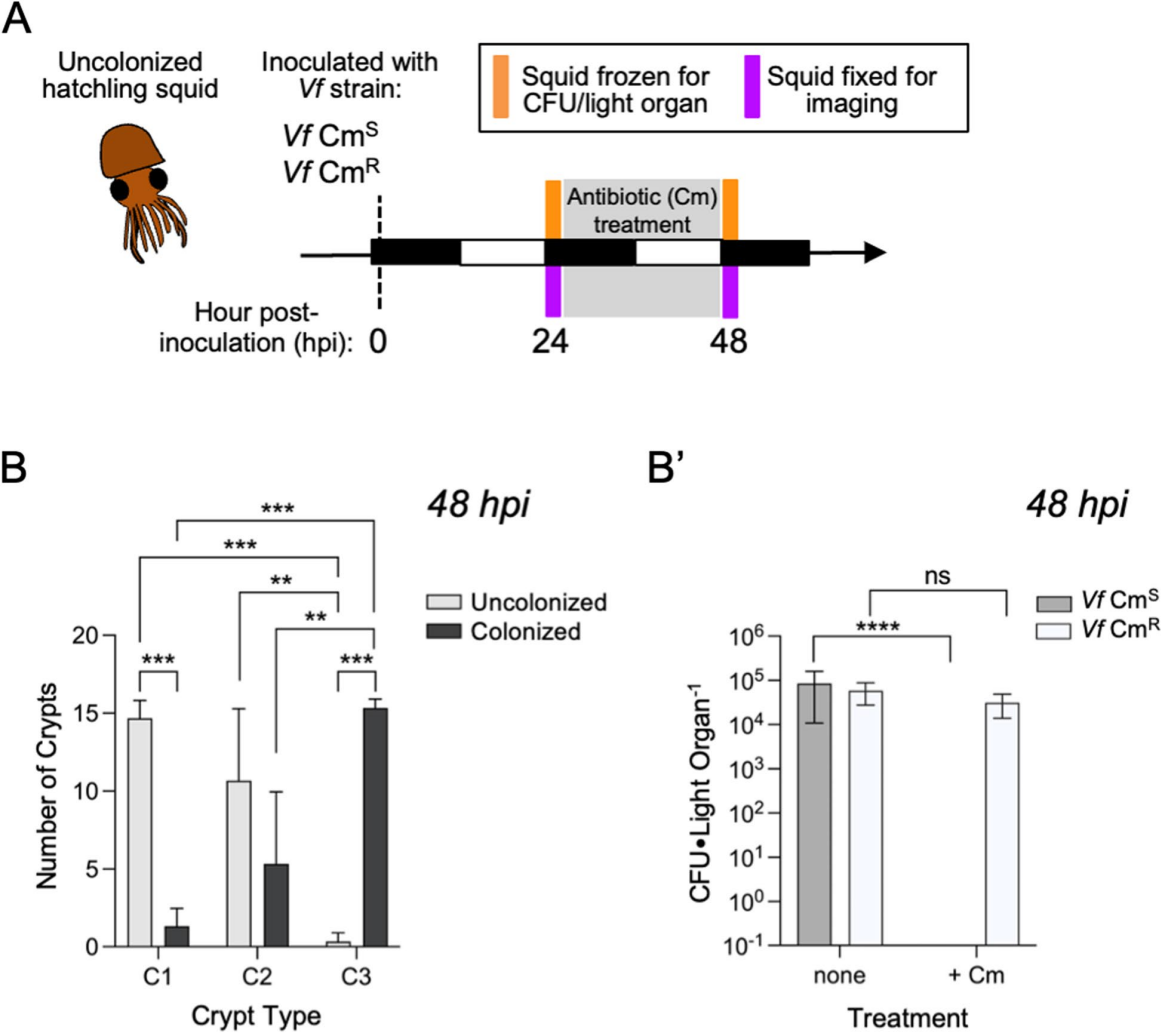
Proportion of *V. fischeri* colonization of each crypt remaining after disturbance by Cm treatment. **A** Schematic of the experimental design and time points corresponding to the day/night cycle (white/black bars, respectively). **B** Efficiency of light-organ colonization with the Cm^S^ strain, by crypt type (C1-C3), after Cm exposure. Colonization status was scored by confocal microscopy as $+ \mathrm{or} -$ ($+$= colonization by as few as 100 cells, $-$ = zero cells) (*n* = 16 lobes, or 8 animals per treatment for each of three clutches; total *n* = 48 lobes, 24 animals). A two-way ANOVA was used to analyze the colonization efficiency for each crypt following Cm treatment F_(2,12)_ = 41.7, *P* $<$ 0.0001. The interaction of crypt type and colonization status explained 87% of the total variation. Asterisks indicate significance determined by Tukey’s multiple comparison test as follows: *** *P* $<$ 0.001, ** *P* $<$ 0.01. **B’** Symbiont population level (average CFU light organ^−1^) at 48 hpost-inoculation (hpi) for the Cm^S^ and Cm^R^ strains after 50 μM Cm treatment (*n* = 6 animals for each of 5 clutches; total *n* = 30). A two-way ANOVA was used to analyze the effect of Cm treatment and strain type on the symbiont load. Cm treatment explained 18% of the total variation, F_(1,78)_ = 22.8, *P* $<$ 0.0001 and the interaction explained 4.8% of the total variation, F_(1,78)_ = 6.2, *P* = .015). Asterisks indicate significant differences as determined by Sidak’s multiple comparison test, **** *P* $<$ 0.0001, ns = not significant

To define the extent of resilience of symbionts in the light organ crypts to antibiotic perturbation, we first determined the resistance of wild-type, chloramphenicol-sensitive (Cm^S^) *V. fischeri* cells to antibiotics under culture conditions. Compared to the chloramphenicol-resistant (Cm^R^) strain, the Cm^S^ strain showed significant growth inhibition at antibiotic concentrations as low as 0.8 μM (~ 0.3 μg mL^−1^) (Fig. 2A), whether the antibiotic was introduced at the beginning of growth, or at mid-log phase (Fig. 2B). In contrast, using both host luminescence output (Fig. 2C) and counts of *V. fischeri* colony-forming units (CFUs) (Fig. 2C’) as measures, the light-organ symbionts were resistant to antibiotics at concentrations more than twofold higher than those inhibiting the growth of cells in culture; specifically, resistance was noted at 2 μM (0.75 μg mL^−1^) Cm (Fig. 2C’). Further, in two replicate experiments, at a higher dose ($\ge$ 50 μM, or 19 μg mL^−1^), both the levels of animal luminescence and CFUs were reduced to undetectable levels (*n* = 7 animals for each replicate). In contrast, the mean symbiont number in animals colonized by the Cm^R^ strain was not affected by this level of antibiotic treatment (Fig. 3; File S1).

### Symbiont sensitivity to antibiotic pressure is different within the distinct light-organ crypt spaces

Although in the abovementioned experiments, high doses of Cm resulted in no detectable symbionts (Fig. 2C’), when Cm selection was removed for a subsequent 24 h, we detected a rebound in both luminescence and CFUs in 9 out of 12 animals. The population rebounded to an average of 13% of the level found in untreated symbiotic juveniles (6 animals from two clutches, *n* = 12 total) (File S1). These data provide evidence that a residual population of *V. fischeri* cells, while initially unable to produced CFUs, remained viable in the light organ and often would eventually grow to reestablish symbiosis. Further, previous observations by confocal microscopy had shown differences in the presence of symbionts across the different crypt types (i.e., C1-C3) after antibiotic treatment ([50]; Fig. 1C). To examine this phenomenon more deeply, we used confocal microscopy to determine patterns of symbiont presence across the crypts under various antibiotic conditions. Here, we colonized animals with either a Cm^S^ or a Cm^R^ strain and examined the colonization levels of each crypt type after a Cm-induced disturbance (8 animals; 16 lobes per treatment per replicate, 3 replicates, *n* = 48 total crypt sets) (Fig. 3A). Following Cm treatment of animals colonized by the wild-type Cm^S^ strain, observation under confocal microscopy indicated that the vast majority of C3 sites examined still contained symbionts (47 out of 48, with 98% of the colonized having $\ge$ 50% of a normal population level); in contrast, C1 sites were principally uncolonized (44 out of 48, with 0% fully colonized). C2 sites were more similar to C1 (16 out of 48 colonized, with 27% fully colonized) (Fig. 3B; File S1). We confirmed that the highest dose of antibiotic treatment did not noticeably affect the symbiotic population levels in animals colonized with the Cm^R^ strain (Fig. 3B’; File S1). These data indicate that although a 24-h antibiotic treatment resulted in no detectable CFUs in the light organ, symbiont cells were observed by confocal microscopy, and the vast majority were found to be in C3. Thus, the source for the observed 25% rebound of CFUs after lifting the antibiotic selection may be a persistent reservoir population in C3.

We next examined the viability of the bacteria observed in C3 using a dye that recognizes cells with compromised membranes, i.e., cells that are presumed to be dead or dying [50]. Following the same experimental design described previously (Fig. 3A), we examined 8 sets of crypts (8 lobes, 4 animals) after exposure to Cm and enumerated the living cells (GFP-labeled) as compared to the non-viable ones (labeled with the dead-cell stain). Most of the symbionts present in C3 appeared to be alive, with very few dead cells (Fig. S2). The detection of apparently live cells in C3 that did not produce CFUs on nutrient agar suggests that the symbionts in C3 are in an altered metabolic state when under antibiotic pressure.

### Symbionts in crypt 3 (C3) serve as a reservoir population that recolonizes other crypts

**Fig. 4:**
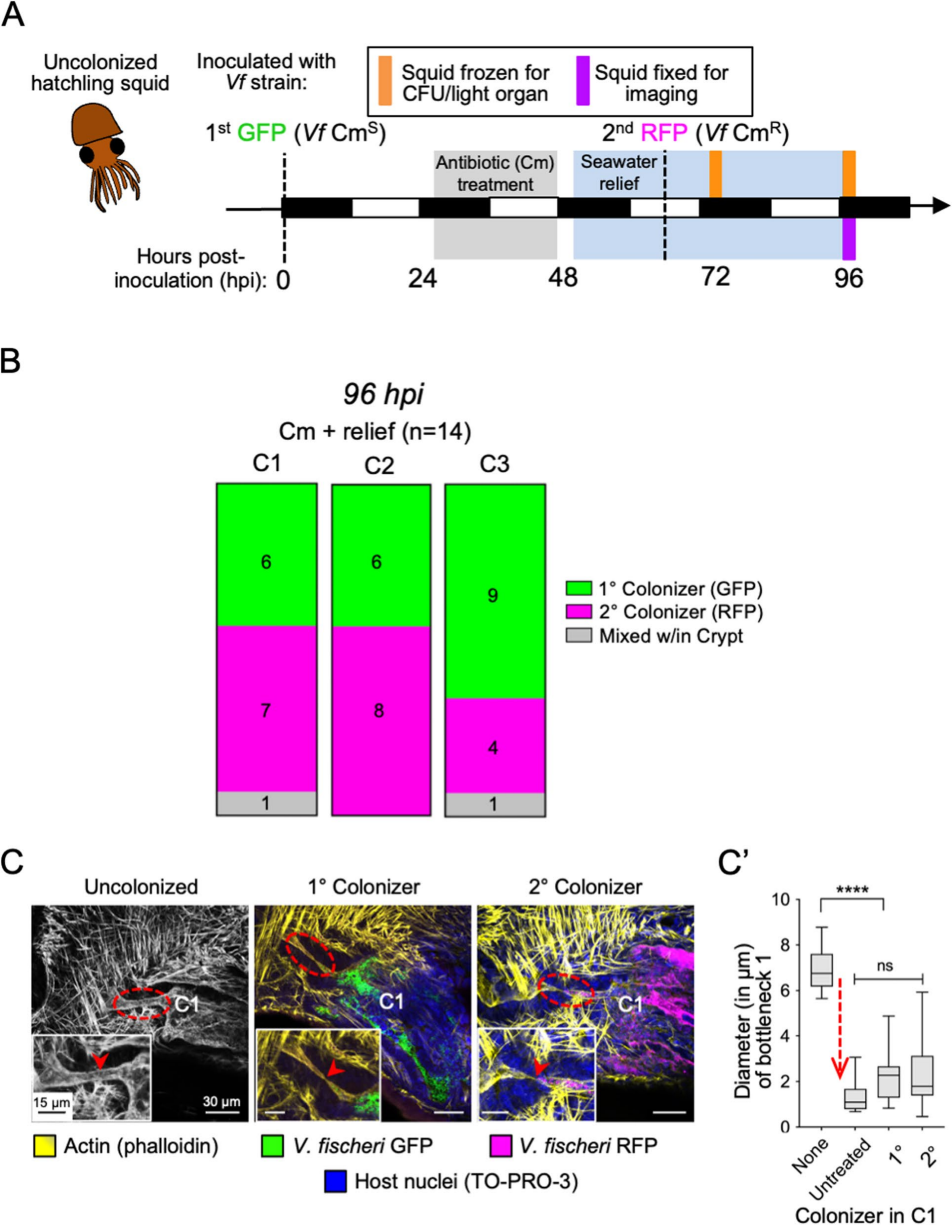
Recolonization of each crypt during recovery from an antibiotic disturbance. **A** Schematic of the experimental design and time points corresponding to the day/night cycle (white/black bars). Dashed lines show the inoculation by the primary symbiont (GFP-labeled strain, *Vf* Cm^S^) and the addition of the secondary symbiont (RFP-labeled strain, *Vf* Cm^R^), added during the seawater relief following 50 μM Cm treatment.** B** The number (and relative proportion) of each crypt type (C1-3) that was occupied at 96 hpi by either the primary colonizer (GFP, green), secondary colonizer (RFP, magenta), or mixed (gray) (*n* = 14 lobes, 7 animals). A *chi*-square test determined that the frequency of C1-3 colonization by primary, secondary, or mixed was not stochastic; $\chi$ ^2^ (4, 14) = 27.6, *P* $<$ 0.01.** C** Confocal micrographs showing bottleneck 1 (BN1; dashed, red circle) when crypt 1 (C1) is uncolonized (left), colonized by either the primary symbiont (GFP, green), or the secondary symbiont (RFP, magenta). Insets show a magnified bottleneck region (red arrowhead). **C’** Measurements of the diameter of BN1 show the constriction phenotype of colonized C1, indicated by red, dashed arrow (*n* = 14 lobes, 7 animals). All colonized C1 groups resulted in a constricted BN1 phenotype (one-way ANOVA; F_3, 45_ = 47.6, *P* $<$ 0.0001). Significance as determined by Tukey’s multiple comparisons test: **** *P* $<$ 0.0001, ns = not significant

With the discovery that C3 remains the most strongly and reliably colonized after antibiotic treatment, we asked whether this crypt could be the principal reservoir of bacteria for the other, symbiont-depleted crypts. Specifically, we compared the potential of symbionts in C3 to repopulate depleted crypts with a competing, externally provided population of the same strain, carrying a different fluorescent marker (i.e., RFP) (Fig. 4A). Because we found that C1 almost never remained colonized after antibiotic pressure (< 1% of the time) (Fig. 3B), only very rarely would any remaining symbionts within C1 be the source for repopulation during recovery (Fig. S3A). Further, the expectation for this experiment would be that if C3 were not the principal source of C1 or C2 repopulation, most of the crypts would be colonized with a mixture of both primary (internal; GFP-labeled) and secondary (external; RFP-labeled) colonizing cells. Instead, mixed crypts were rare (2 out of 42 crypts; one C1 and one C3) (Fig. 4B; File S1). Further, the data show that, after antibiotic pressure is relieved under these conditions, all three crypt types were colonized by one or the other strain in most animals (Fig. 4B). In addition, this colonization of C1 by either the primary or secondary symbiont rescues the normal host phenotype of a constricted bottleneck (Fig. 4C, C’) [50]; this phenotype promotes retention of symbionts [50] and is an indicator of a sizeable population actively engaging in quorum sensing [59].

### Antibiotic disturbance induces venting behavior in animals colonized by a Cm^S^ strain

**Fig. 5:**
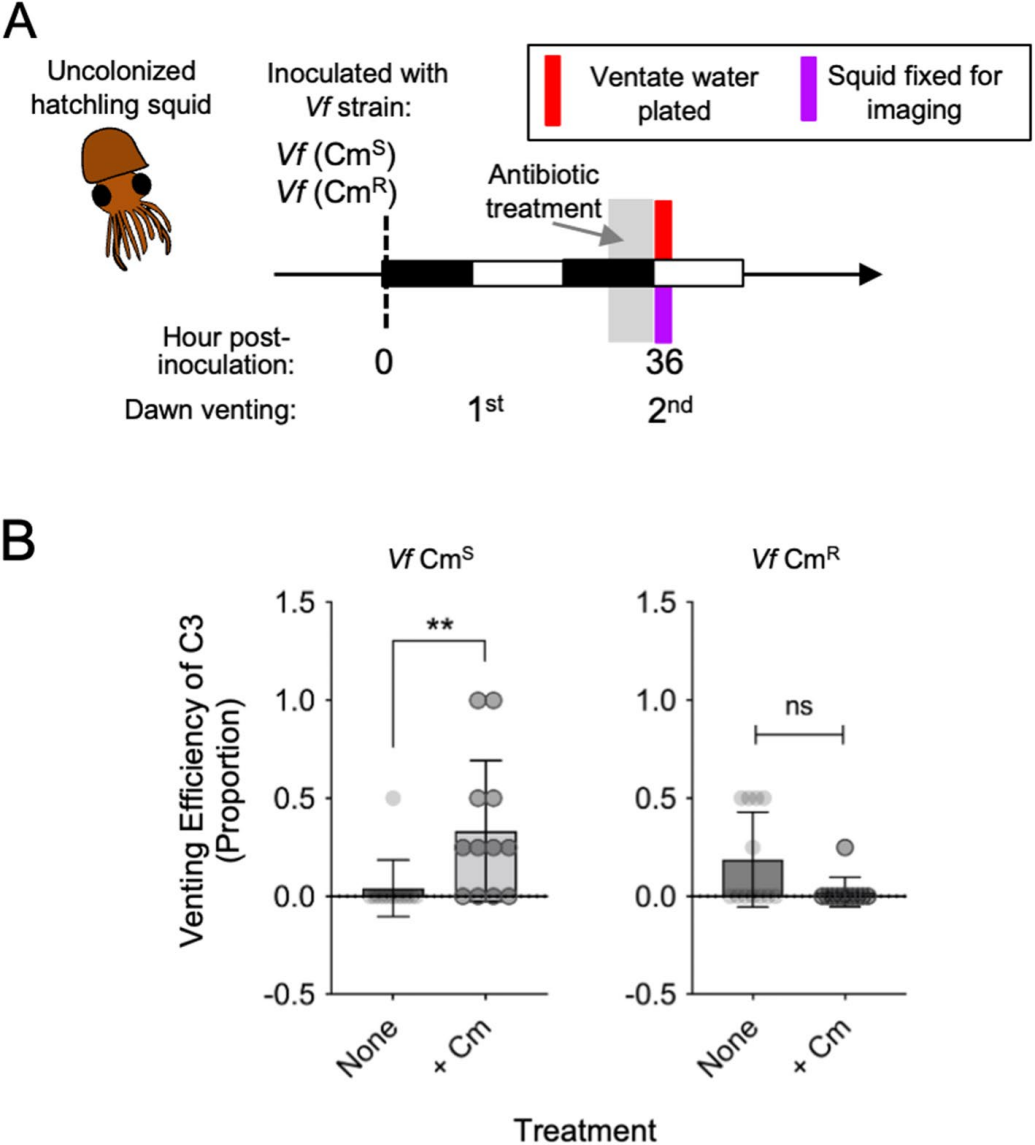
An antibiotic-induced disturbance affects venting behavior of crypt 3 (C3). **A** Schematic of experimental design examining symbiotic squid, colonized by either *Vf* Cm^S^ or *Vf* Cm^R^, that were then incubated with 20 μM Cm for 6 h prior to sampling. **B** Venting efficiency scored for C3 as the proportion of the migration paths that contained *V. fischeri* cells, as determined by confocal microscopy. Cm treatment increased venting in C3 only for *Vf* Cm.^S^ colonized squid. Mann–Whitney *U* test (*n* = 12 lobes, 6 animals per treatment); left, difference in median = 0.25, U = 24.5, *P* $<$ 0.01; right, difference in median = 0, U = 43.5, *P* = 0.0974

Under normal conditions, C3 does not exhibit the typical daily crypt venting until 72 h after colonization [50]. As expected, C3 did not vent at this time without Cm treatment, and this lack of venting was also found for crypts colonized by the Cm^R^ strain under Cm pressure (Fig. 5, Fig S4). This finding suggests that Cm itself does not induce the host to vent; rather, C3 is only induced to vent when a Cm-treated host is colonized by a Cm^S^ strain (Fig. 5B). These data suggest that the Cm-associated changes in host venting behavior result from either an autonomous response of C3, or a sensing by C3 of the Cm-driven changes in C1 or C2.

### The biogeography of the juvenile light organ provides evidence that C3 is the primary source of recolonization after antibiotic disturbance

**Fig. 6:**
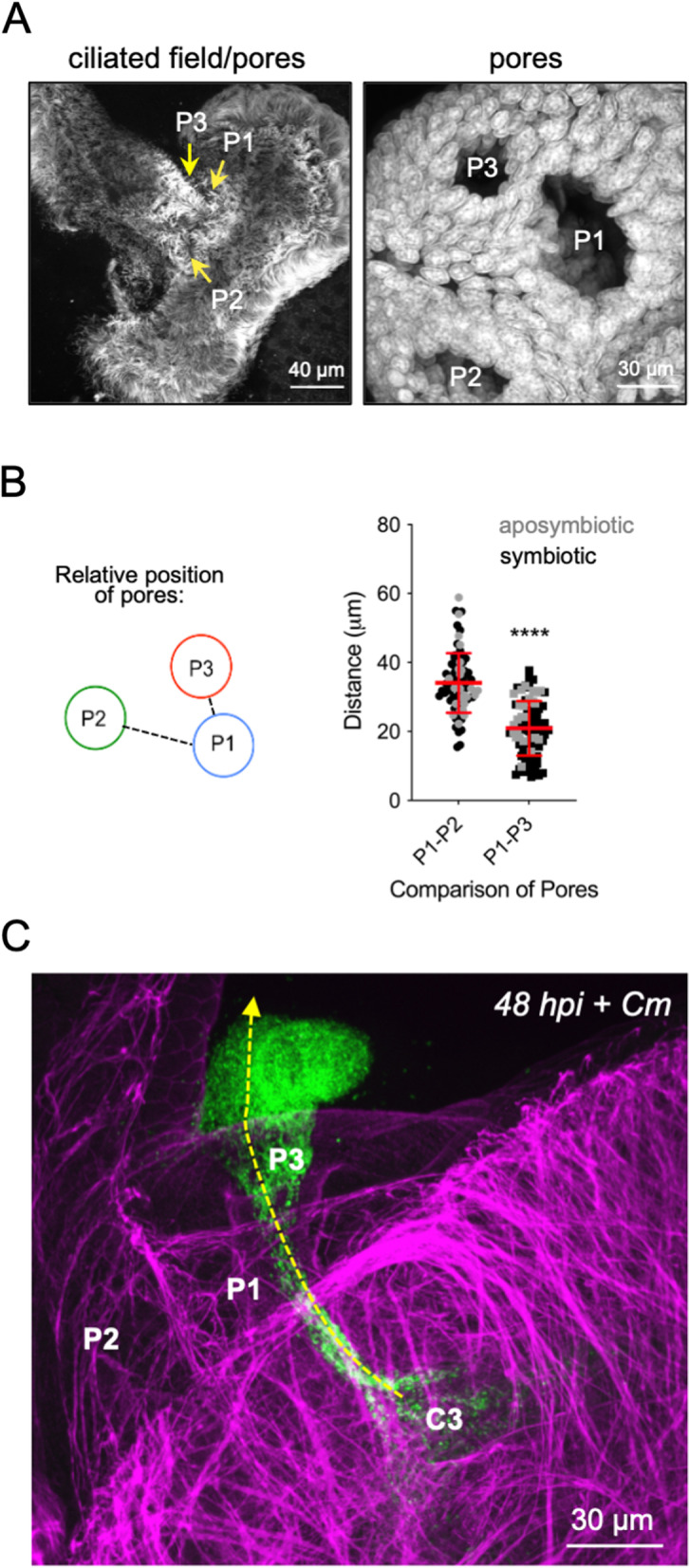
Potential routes of re-infection after antibiotic disturbance. **A** Confocal micrographs revealing the relative position of each pore on the superficial epithelium of the light organ. *Left*, ciliated epithelium (anti-acetylated $\alpha$-tubulin antibody); *Right*, nuclei of cells surrounding the pores (TO-PRO-3). **B** *Left*, diagram showing the typical positioning of the pores. *Right*, distance between pore 1 and each of the other pores, revealing a shorter distance between P1 and P3. This distance was 13.14 μm less than between P1 and P2; paired *t* test (*T* = 10.51, df = 80, *P* $<$ 0.0001). **C** Confocal micrograph illustrating expulsion of *V. fischeri* (green) from crypt 3 (C3) after Cm treatment, and symbiont cells “hovering” at the surface near the opening of P1 (see Movie S1)

We measured the position of superficial pores on each lobe relative to one another to determine whether their relationship may contribute to the process of recolonization (Fig. 6). The data show that typically the distance between pore 1 (P1) and pore 3 (P3) is about half the distance between P1 and pore 2 (P2) (Fig. 6A, B). These data suggest that even if C2 has residual symbionts after antibiotic pressure, repopulation via P1 will be more likely by symbionts venting from P3 (Fig. 6C, Movie S1).

## Discussion

This study provides insight into how a symbiosis recovers from a chemical disturbance through a repopulation by symbionts from a protected microenvironment within the host. Using a binary model of symbiosis, the squid-vibrio system, we examined how the sequential maturation and different morphology of the host’s crypt tissues generates the potential for a disturbance-resistant internal reservoir of symbionts. This reservoir can perform two functions: retaining bacteria during an antibiotic disturbance, and reseeding the more mature antibiotic-cleared sites in the host organ, creating a strategy that provides resiliency to the association. Resiliency of a host-microbe partnership, especially during the early, stress-susceptible stage of life, is critical for the health of the microbiome and, ultimately, for the host. Further, similar reservoir sites within symbiotic tissues of other hosts may be integral to recovery from disturbances by similarly providing a source of microbes that ensures a successful, persistent symbiosis or microbiome.

Characteristics of both partners of the squid-vibrio symbiosis are likely to contribute to the reservoir function of the crypt 3 (C3) colonization site. One hypothesis for why *V. fischeri* cells tolerate the disturbance of antibiotic treatment while occupying C3 is that this symbiont subpopulation, like those in other symbioses, experiences different conditions that impact their physiology and growth [60, 61]. Such differences in the growth state of the bacteria could explain why the bacteriostatic antibiotic used, one that targets bacterial protein synthesis, did not have the same effect in C3 as it did in C1. While the timing of colonization is similar for all three crypt-types (C1-C3), the physiology and behavior of the symbiont population in C3 are different. For example, symbionts in C3 have delayed light production [48], are a greater distance from the apical surface of the crypt epithelia, and normally exhibit an increased prevalence of dead cells at the periphery of the population [50]. Taken together, such symbiont-related factors may all contribute to the first function of the reservoir, i.e., for a subset of the bacteria to be in a physiological state that can better withstand stresses like an antibiotic disturbance. Future work will be directed towards characterizing the differences in physicochemical features between each crypt that enable symbionts to differentially withstand antibiotic-induced and other disturbances.

Host-related factors likely also contribute to the functionality of the C3 reservoir, including differences in the crypt morphology, bottleneck constriction, and venting behavior [50]. The distinct morphology of C3 at hatching may play a direct role in its reservoir function by shaping the physicochemical environment. Evidence suggesting that the specific morphology of C3 is linked to a distinct physicochemical environment includes that the least mature stages of C3 have a higher prevalence of dead symbionts, indicating a fitness cost for symbionts to colonize underdeveloped crypts [62]. In addition, the epithelial cells comprising C3 do not respond to the presence of symbionts with the same increase in cytoplasmic volume and cuboidal shift as is seen in C1 [63]. It is not yet known whether the absence of these symbiotic responses by C3 epithelium is due to physical barriers preventing bacterial cues from reaching the host, or to the inability of these host cells to respond in the same way as the more mature tissues of C1. Finally, in the present study, we considered the biogeographic placement of the pores and suggested that the proximity between P3 and P1 enhances the capacity for cross-crypt recolonization after antibiotic disturbance.

Two other distinguishing characteristics of C3 are the degree of its bottleneck constriction and its lack of venting behavior. The bottleneck connected to C3 (BN3) is less discriminating to the activity of the symbionts within C3, both in terms of signaling and light production. In contrast, BN1 is highly discerning and does not constrict when symbionts lack normal quorum-signaling activity, even in the presence of light production [59]. These differences in gatekeeper functionality may result in varied levels of selectivity for symbionts that have a high fitness, at least in terms of an ability to supply the host with luminescence. Even more striking is that the early lack of venting from C3 means that the bacteria that colonize it may not experience the daily changes in population density, and perhaps nutrient turnover, in the same way as symbionts in C1. The finding that C3 could be induced to vent when an antibiotic treatment coincided with the dawn light cue indicated that the system reacts to a disturbance. Previous work demonstrated that a “cheater” strain of *V. fischeri*, i.e., one that does not luminesce, can be detected in C3 even after it was outcompeted by the wild-type strain in C1, and that this retention in C3 may be a mechanism by which less fit strains can persist at low levels within the light organ [51, 56]. Taken together, this combination of factors, along with the findings presented here, suggests that the different crypt maturation states provide a diversity of microhabitats at hatching that may result in different interactions with the bacterial symbionts that colonize them.

The goal of this study was to leverage the squid-vibrio system to examine how spatial heterogeneity and state of maturity of host crypts work to promote a stable partnership with environmentally acquired bacteria. Several studies have been done on the role of the microbiome in gut development of vertebrates and invertebrates [64]; however, the mechanisms underlying resiliency of the juvenile microbiome remain poorly understood. Extensive selection pressure has resulted in both host and symbiont factors that enable the acquisition of environmental microbes anew at each generation of the host; such horizontally transmitted symbioses require biochemical and biophysical features of both partners to initiate a partnership [39, 44, 65]. Yet, even after a symbiosis is initiated, its stability is vulnerable to disturbances, which can eliminate symbionts from certain tissue sites, or from the host entirely. These disturbances can be host-driven, such as the peristaltic movements in gut tracts of mammals [66], fish [67], and insects [68], or the venting behavior that expels symbionts from the squid light organ [69]. External or environmental disturbances can include phage predation on symbionts, exposure to antibiotics, or changes in temperature, salinity, or pH, especially for marine invertebrates [70–75]. Such disturbances may routinely threaten the homeostasis of a symbiotic community throughout the host’s lifespan. As such, an internal reservoir would both maximize the chances of a successful recovery and serve as an “insurance policy” for the host. We propose that the spatial heterogeneity of host tissues, which establishes a biogeography that shapes the initial colonization of a host, may also impact symbiotic recovery after perturbation. The current study presents a model to test a “strategy” by which host tissues better ensure a persistent symbiosis, even when the bacteria are at low in abundance in the environment and may be difficult to recapture after a certain developmental stage. Clearly, an effort to determine the extent to which specific tissue sites serve as an internal reservoir of horizontally acquired symbionts in other animals requires further attention.

The colonization of the host epithelium in the light organ has parallels with the early development of the human gut microbiome. One such parallel is a limited diversity and the importance of the early colonizers in the trajectory of the development of the microbiome [76]. For example, in the infant gut, the microbiome is initially dominated by the genus *Bifidobacterium*, but diversifies over time [77]*.* Postnatal antibiotic treatment dramatically shifts the community structure by decreasing the prevalence of *Bifidobacterium* and increasing the abundance of *Klebsiella* and *Enterococcus* spp. [78]. This shift is highly deleterious to the infant host because *Bifidobacterium* and other typical early colonizers are critical not only to initiating the proper succession of microbiome development, but also to driving the maturation of host tissues and their immune function [79]. While the microbial balance in early life affects developmental trajectories, and later health outcomes, the mechanisms by which a developing host’s microbiome recovers from possible disturbances are not fully understood [80, 81].

The second important parallel between the squid and mammalian systems is that bacterial symbionts may reside in protected microenvironments within the tissues; however, whether these bacteria are a source for recolonization is not yet known. Recent work has underscored how environmental populations can be external sources of symbionts that rebalance dysbiotic communities following an environmental disturbance [21, 37, 82]. However, identifying internal sources of symbiont recolonization in mammals is crucial to understanding how the gut microbiome recovers after antibiotic-induced dysbiosis. Studies of symbiont-host interactions across gut-tissue biogeography have shown that a single species, *Bacteroides fragilis*, is permitted to colonize protected niches in the crypt spaces of the mammalian colon [83]. The resident microbiota of these mucosal crypts [84, 85] have been proposed as an internal reservoir that may prevent antibiotic-induced dysbiosis by providing a source of protected *B. fragilis* symbionts [86]. A second potential reservoir is the vermiform appendix, which may have dual functions in both reseeding proximal portions of the gut tract, and serving as a key site for the development of the immune system [87–89]. Future work investigating the extent to which these and other potential reservoirs function to reseed host tissues following a disturbance will provide a needed understanding of how these partnerships are maintained, and will inform the development of interventions to treat dysbiotic microbiomes [90].

## Conclusion

Colonization by microbes in early life has both immediate and long-term impacts that benefit host organisms. As such, acquiring and maintaining the right microbial partners across key developmental stages and disturbances is vital for host health and maturation. The findings presented here show that variations in tissue biogeography create a functional reservoir that aids in microbiome stability and symbiotic resiliency. Identifying the conserved strategies of host-microbe partnerships across phyla will illustrate fundamental themes in the evolution and function of these important symbioses.

## Supplementary Information

**Additional file 1: Movie S1.** Video of optical sections taken by confocal microscopy at 48 hpi following treatment with Cm, illustrating the expulsion of *V. fischeri* Cm^S^ (GFP, green) from C3, which is just above P1 in this image (F-actin, magenta; TO-PRO-3, blue).

**Additional file 2: Fig. S1.** Growth curves for *V. fischeri* strains, *Vf* Cm^S^ (**A**) and *Vf* Cm^R^ (**A’**), in response to continuous exposure to Cm in SWT medium.

**Additional file 3: Fig. S2.** Symbiont viability in C3. Counts of live or dead cells present in crypt 3 at 48 hpi following treatment with 50 μM Cm. A Mann-Whitney U test was used to compare the mean rank of live cells (12.19) to the mean rank of dead cells (4.81), U = 2.5, *P<*0.01, as indicated by the asterisks*.*

**Additional file 4: Fig S3.** Crypt 3 (C3) as the principal reservoir of symbionts. **(A) **Colonization efficiency at 72 hpi by crypt (C1-C3) for each strain, as scored by confocal microscopy. Colonization of C1 and C2 remains low after relief from Cm treatment, whereas symbiont occupation of C3 is still high. A two-way ANOVA was used to analyze the effect of Cm treatment on each strain as a function of crypt type. The strain type (Cm-sensitive or -resistant) explained 57% of the total variation (*F *_3, 150_ = 57.2, *P* $<$ .0001). Asterisks indicate significance determined by Tukey’s multiple comparison test as follows: **** *P* $<$ .0001, ** *P* $<$ .01 (*n* = 18 lobes, 9 animals per treatment for a single clutch). **(B)** Symbiont number (CFU per light organ) for each strain of symbiont at 96 hpi (*n* = 5 animals per treatment). **(C)** Luminescence output of animals at 96 hpi (*n* = 10 animals for Apo and Sym controls, n = 14 animals for Cm treatments). A one-way ANOVA was used (*F*_3, 44_ = 11.4, *P* $<$ 0.0001). Asterisks indicate significance determined by Sidak’s multiple comparison test as follows: **** *P* < 0.0001, ** *P* $<$ 0.01, * *P* $<$ 0.05.

**Additional file 5: Fig S4. **Luminescence response of symbionts to Cm treatment during venting. Cm treatment reduces luminescence output only for animals colonized by the Cm^S^ strain of *V. fischeri*. Groups were compared by a one-way ANOVA (F_2,__34_ = 76.6, *P* $<$ 0.0001). Asterisks indicate significance from Tukey’s post-hoc test: **** *P* $<$ 0.0001.

**Additional file 6. **Summary Tables for Crypt 3 paper.

## Data Availability

All data generated or analyzed during this study are included in the published article and its supplementary information files.

## Abbreviations

Apo: Aposymbiotic
BN1: Bottleneck 1
BN3: Bottleneck 3
C1: Crypt 1
C2: Crypt 2
C3: Crypt 3
CFU: Colony-forming unit
Cm: Chloramphenicol
Cm^S^: Sensitivity to Cm
Cm^R^: Resistance to Cm
GFP: Green fluorescent protein
Kn: Kanamycin
hpi: Hours post-inoculation
LU: Light units
ns: Not statistically significant
P1: Pore 1
P2: Pore 2
P3: Pore 3
Sym: Symbiotic
RFP: Red fluorescent protein
*Vf*: *Vibrio* *fischeri*
