# Supplementary figures and images for: Maturation state of colonization sites promotes symbiotic resiliency in the Euprymna scolopes-Vibrio fischeri partnership
Source: Microbiome. 2023 Mar 31;11:68. doi: 10.1186/s40168-023-01509-x (PMC10064550; doi:10.1186/s40168-023-01509-x)

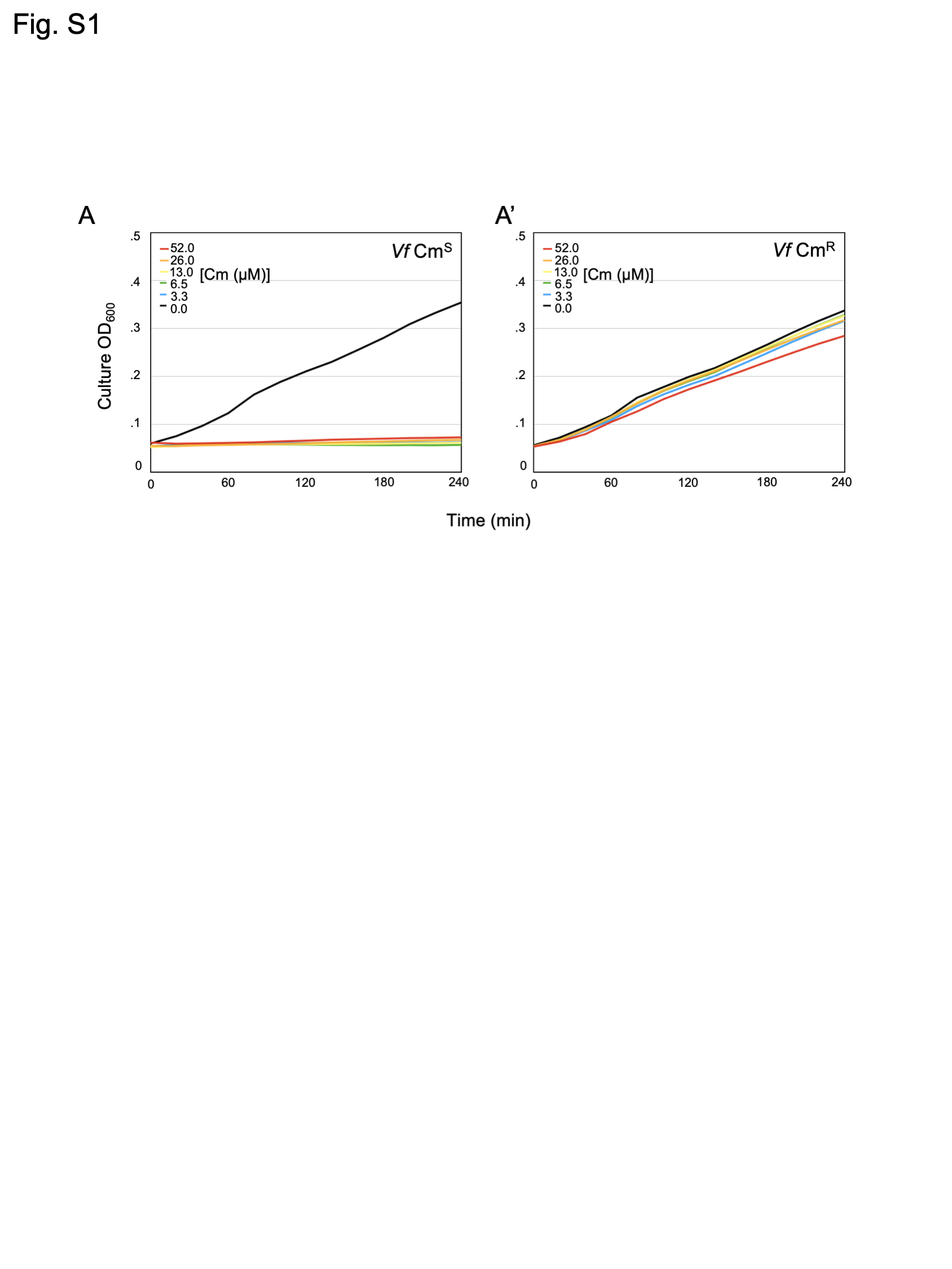

Supplement: Supplementary file 3 — Additional file 2: Fig. S1. Growth curves for V. fischeri strains, Vf CmS (A) and Vf CmR (A’), in response to continuous exposure to Cm in SWT medium. [file 40168_2023_1509_MOESM2_ESM.tiff]

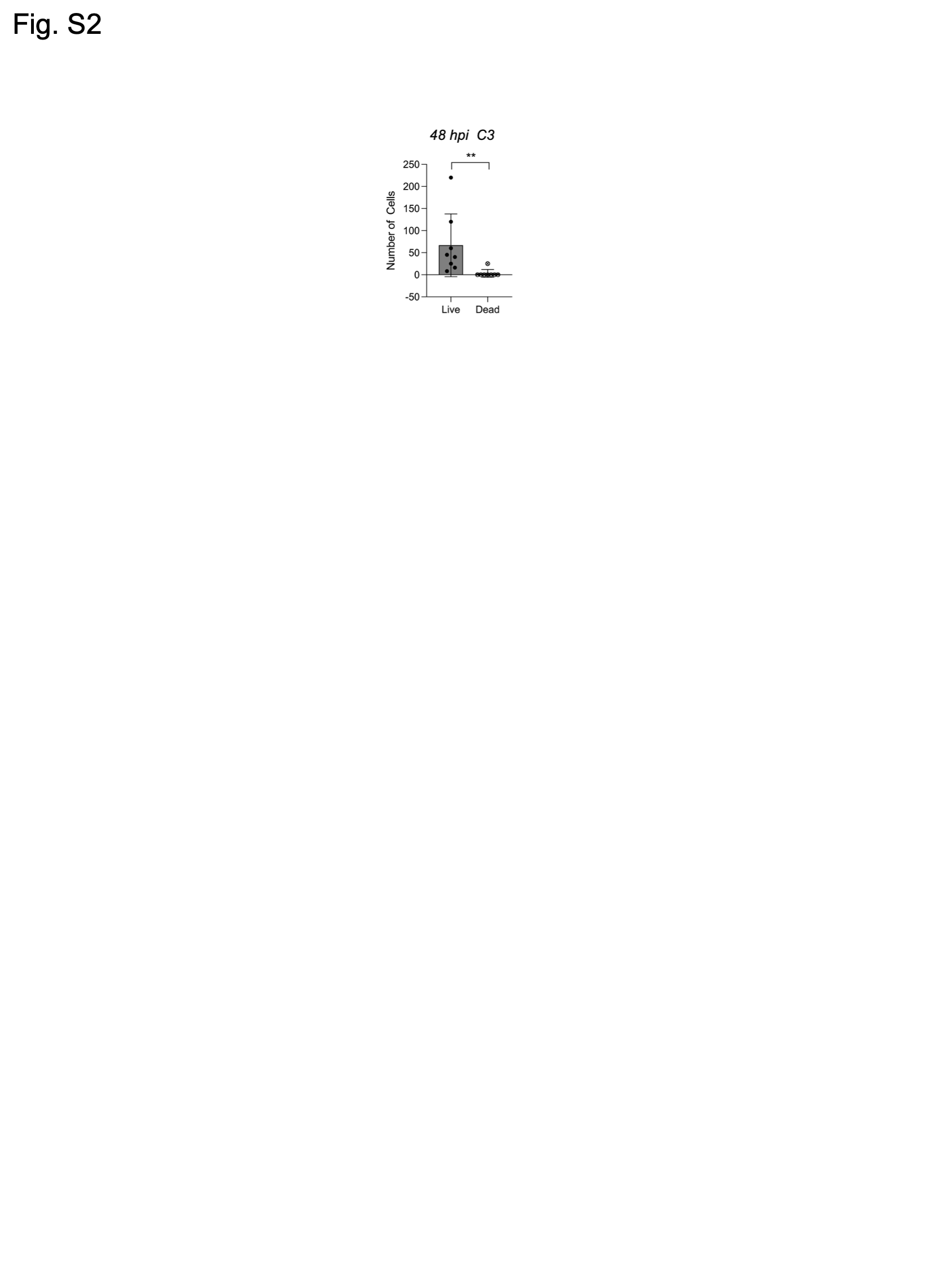

Supplement: Supplementary file 4 — Additional file 3: Fig. S2. Symbiont viability in C3. Counts of live or dead cells present in crypt 3 at 48 hpi following treatment with 50 μM Cm. A Mann-Whitney U test was used to compare the mean rank of live cells (12.19) to the mean rank of dead cells (4.81), U = 2.5, P<0.01, as indicated by the asterisks. [file 40168_2023_1509_MOESM3_ESM.tiff]

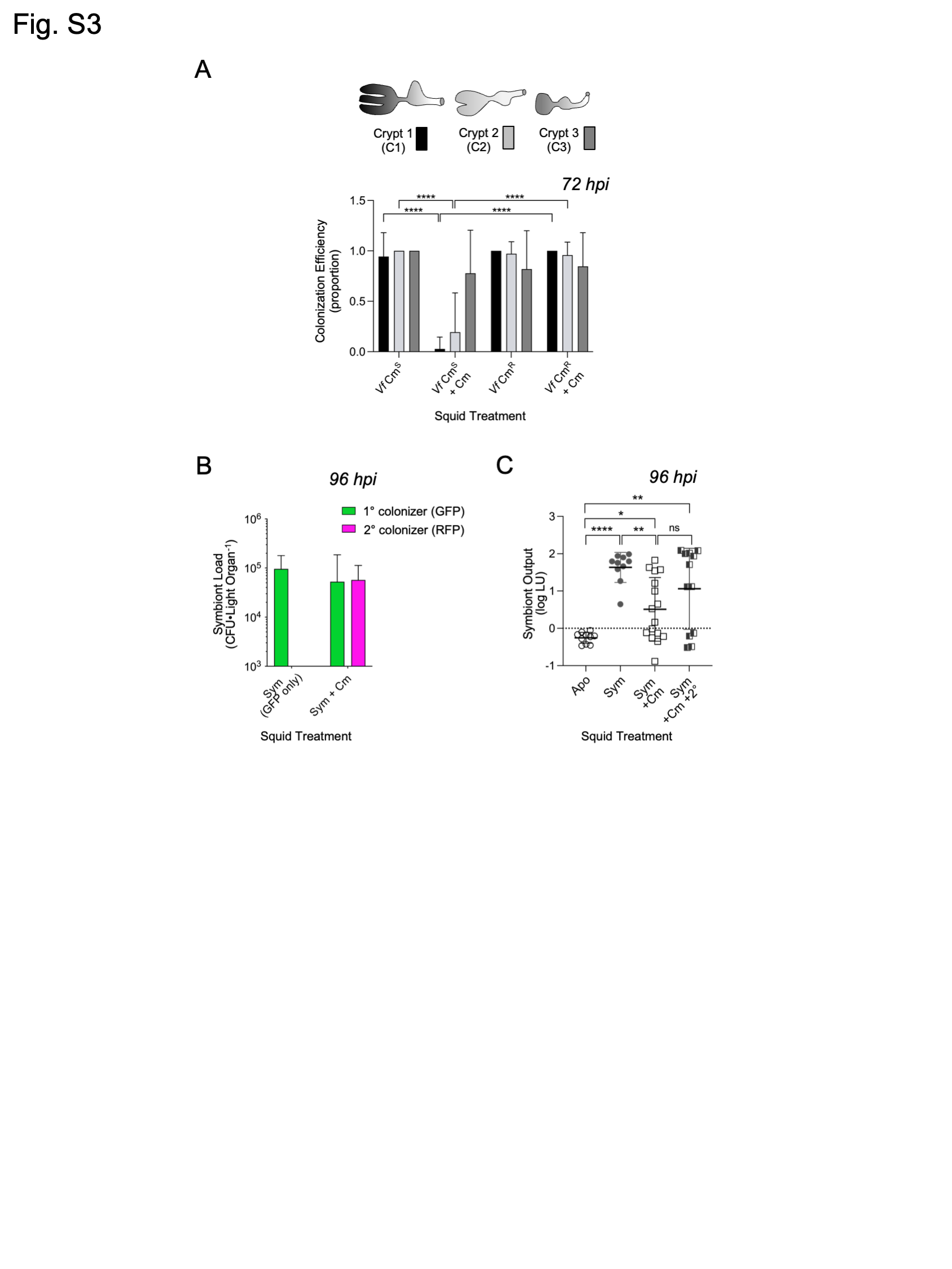

Supplement: Supplementary file 5 — Additional file 4: Fig S3. Crypt 3 (C3) as the principal reservoir of symbionts. (A) Colonization efficiency at 72 hpi by crypt (C1-C3) for each strain, as scored by confocal microscopy. Colonization of C1 and C2 remains low after relief from Cm treatment, whereas symbiont occupation of C3 is still high. A two-way ANOVA was used to analyze the effect of Cm treatment on each strain as a function of crypt type. The strain type (Cm-sensitive or -resistant) explained 57% of the total variation (F 3, 150 = 57.2, P $<$< .0001). Asterisks indicate significance determined by Tukey’s multiple comparison test as follows: **** P $<$< .0001, ** P $<$< .01 (n = 18 lobes, 9 animals per treatment for a single clutch). (B) Symbiont number (CFU per light organ) for each strain of symbiont at 96 hpi (n = 5 animals per treatment). (C) Luminescence output of animals at 96 hpi (n = 10 animals for Apo and Sym controls, n = 14 animals for Cm treatments). A one-way ANOVA was used (F3, 44 = 11.4, P $<$< 0.0001). Asterisks indicate si [file 40168_2023_1509_MOESM4_ESM.tiff]

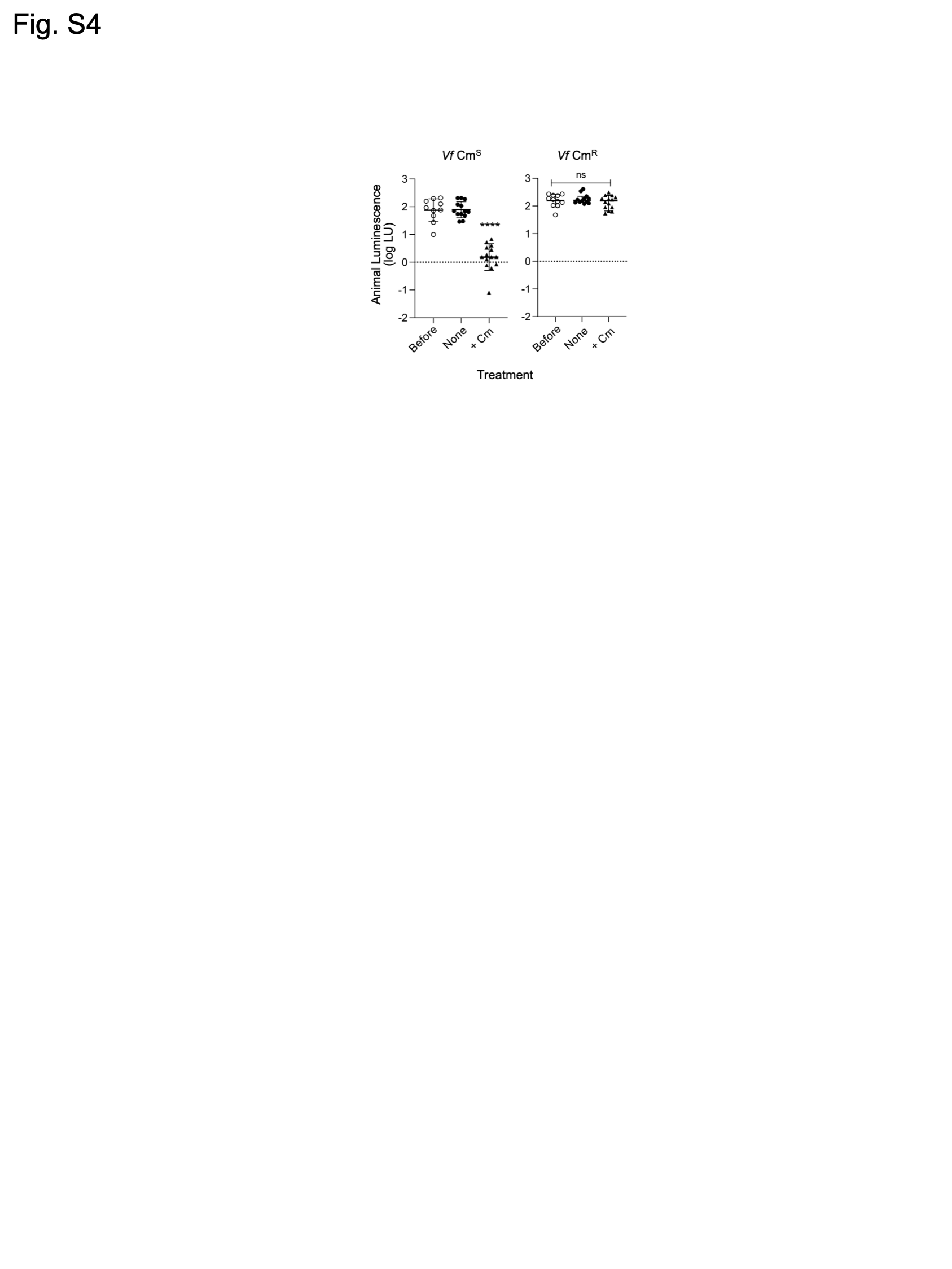

Supplement: Supplementary file 6 — Additional file 5: Fig S4. Luminescence response of symbionts to Cm treatment during venting. Cm treatment reduces luminescence output only for animals colonized by the CmS strain of V. fischeri. Groups were compared by a one-way ANOVA (F2, 34 = 76.6, P $<$< 0.0001). Asterisks indicate significance from Tukey’s post-hoc test: **** P $<$< 0.0001. [file 40168_2023_1509_MOESM5_ESM.tiff]
